# Changes in nailfold videocapillaroscopy in patients with granulomatosis with polyangiitis (Wegener’s): an observational study

**DOI:** 10.1186/1479-5876-10-S3-P29

**Published:** 2012-11-28

**Authors:** Julia Uceda, Rosalía Martinez, María L Velloso, José L Marenco

**Affiliations:** 1 Rheumatology Dept., Valme University Hospital, Sevilla, Spain

## Background

Nailfold videocapillaroscopy (NFC), allows for the detection of changes in microcirculation. In the granulomatosis with polyangiitis (GPA) the existence of a defined pattern has not been found.

## Objectives

The main objective of our study was to detect the possible existence of a defined pattern in the microcirculation of the nailfold capillaries of patients with GPA. The second objective was to investigate the possible correlation between abnormalities found and systemic involvement.

## Methods

We identified 10 patients with a current mean age of 55.7 ± 16.5 years and predominantly female (60%). The mean age at diagnosis was 49.4 years. 70% had upper respiratory tract involvement, the same percentage had pulmonary involvement (cavitated nodules or alveolar hemorrhage), the cutaneous manifestations such as purpura or necrotic ulcers were present in 70%. About 40% had renal involvement (renal failure, proliferative glomerulonephritis), and 40% had peripheral neurological involvement. NFC was carried out by the same rheumatologist, on fingers 3 through to 5 of both hands using a ZUZI videocapillaroscopy, trinocular, dual illumination and zoom of 1 X 4 X.

## Results

Abnormalities of the microcirculation of nailfold capillaries were found in 8 of the 10 patients. Among the patients with this pathological microcirculation, 62.5% had structural alterations (tortuous capillaries), 50% presented with micro-hemorrhage (single or multiple), avascular areas were found in 37.5% and 75% showed lower capillary density. Neither capillary dilation nor the formation of new vessels were detected within the sample of patients. Next table correlates capilaroscopic finding with organ involvement.

## Conclusions

We have observed, more frequent bleeding, avascular areas and reduced capillary density and these findings were not related with any specific organ involvement. There is one only study in GPA which communicates a high percentage of avascular areas.
